# An Antibody to De-N-Acetyl Sialic Acid Containing-Polysialic Acid Identifies an Intracellular Antigen and Induces Apoptosis in Human Cancer Cell Lines

**DOI:** 10.1371/journal.pone.0027249

**Published:** 2011-11-09

**Authors:** Lindsay M. Steirer, Gregory R. Moe

**Affiliations:** 1 Centers for Cancer, Children's Hospital Oakland Research Institute (CHORI), Oakland, California, United States of America; 2 Immunobiology and Vaccine Development, Children's Hospital Oakland Research Institute (CHORI), Oakland, California, United States of America; Faculdade de Medicina, Universidade de São Paulo, Brazil

## Abstract

Polysialic acid (PSA), an α2,8-linked homopolymer of N-acetylneuraminic acid (Neu5Ac), is developmentally regulated and its expression is thought to be restricted to a few tissues in adults. Recently, we showed that two human pathogens expressed a derivative of PSA containing de-N-acetyl sialic acid residues (NeuPSA). Here we show that an epitope identified by the anti-NeuPSA monoclonal antibody, SEAM 3 (SEAM 3-reactive antigen or S3RA), is expressed in human melanomas, and also intracellularly in a human melanoma cell line (SK-MEL-28), a human T cell leukemia cell line (Jurkat), and two neuroblastoma cell lines (CHP-134 and SH-SY5Y). SEAM 3 binding induced apoptosis in the four cell lines tested. The unusual intracellular distribution of S3RA was similar to that described for the PSA polysialyltransferases, STX and PST, which are also expressed in the four cell lines used here. Interestingly, suppression of PST mRNA expression by transfection of SK-MEL-28 cells with PST-specific short interfering RNA (siRNA) resulted in decreased SEAM 3 binding. The results suggest further studies of the utility of antibodies such as SEAM 3 as therapeutic agents for certain malignancies.

## Introduction

PSA modification appears to be limited to a few animal proteins and the capsular polysaccharides of the neuroinvasive bacteria *Neisseria meningitidis* group B (NmB) and *E. coli* K1 [1]. In humans, PSA has been shown to be present on neural cell adhesion molecule (NCAM) [2], synaptic cell adhesion molecule 1 [3], the alpha subunit of the voltage sensitive sodium channel [4], the integrin alpha 5 subunit [5] the scavenger receptor CD36 [6], neuropilin-2 [7], and the PSA polysialyltransferases ST8Sia2 and ST8Sia4, also known as STX and PST, respectively [8]. NCAM is the most abundant polysialylated protein, especially during fetal development, and the role of polysialylation in NCAM function is the most thoroughly investigated [9]. NCAM polysialylation blocks the adhesive properties of NCAM to allow cell migration and modulate other NCAM functions [9]. In adult mice, PSA expression is limited to a few tissues in the brain that exhibit synaptic plasticity such as the olfactory bulb and hypothalamus [9] and may have a role in T cell development [10]. Some human tumors, including astrocytoma [11], small cell and non-small cell lung carcinoma [12], multiple myeloma [13], neuroblastoma [14], rhabdomyosarcoma [15] and Wilms' tumor [16] express PSA and the relative level of PSA expression in some cancers has been associated with poor prognosis [12], [17].

During the development of vaccines for the prevention of disease caused by NmB, we discovered that a murine monoclonal antibody (mAb) SEAM 3, which was produced by immunization with an N-propionyl derivative NmB capsular polysaccharide (N-Pr MBPS)-based vaccine [18], recognized PSA antigens that contained de-N-acetylated neuraminic acid (Neu) residues [19], [20], [21]. The presence of Neu residues in the N-Pr MBPS-tetanus toxoid vaccine was an unintended side product resulting from incomplete re-N-acylation. Subsequently, we showed that neuraminic acid-containing PSA (NeuPSA), including fully de-N-acetylated PSA, was immunogenic and elicited antibodies that were protective against NmB and NmC strains [19].

Although NeuPSA had not been described previously in humans, shorter Neu-containing sialic acid antigens (NeuSia), such as gangliosides had been reported to be present in some human tumors and cancer cell lines [22], [23], [24]. NeuSia antigens expressed in human tumors include NeuSia variants of the mono- and disialylogangliosides GM3 and GD3 [22], [23], [24], [25], [26], respectively. Hakomori and coworkers showed that NeuSia GD3 expressed in the human epithelial carcinoma cell line A431 was a strong activator of epidermal growth factor receptor kinase in Triton X-100-treated cells [27], [28]. The result suggests that the NeuSia GM3 derivative may have a role in activating receptor pathways that promote cell proliferation. Using radioactive labeling experiments in melanoma cell lines, Varki and coworkers showed that N-acetyl groups in gangliosides GD3 and GM3 turned over more rapidly than the parent molecules, suggesting the existence of NeuSia-containing gangliosides in these cells [25]. Recently, Popa et al isolated and structurally characterized NeuSia-containing GD3 from primary human melanoma tumors [26]. Since some cancer cells express NeuSia antigens, it raised the question of whether the longer NeuPSA-modified antigens are also expressed by human cancer cells. In the following, we investigated the reactivity of SEAM 3 with normal human skin, primary human melanoma tumors and several human cancer cell lines including leukemia, melanoma, and neuroblastoma cells, and the functional activity of SEAM 3 against these cancer cell lines.

## Results

### Specificity of mAb SEAM 3

**Figure 1:**
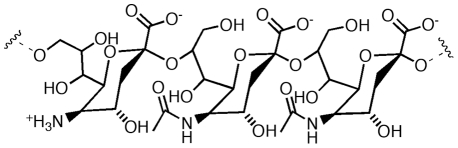
Minimal NeuPSA epitope recognized by SEAM 3 as determined by inhibition ELISA with NeuPSA derivatives (**Table 1**).

We have previously shown that SEAM 3 is reactive with a variety of Neu-containing oligosialic acid (OSA)/PSA derivatives [18], [19], [20]. To define the specificity of SEAM 3 with respect to NeuOSA/PSA antigens likely to be expressed naturally (ie. N-acetyl-containing derivatives), we prepared partially de-N-acetylated derivatives of OSA by mild base treatment of purified oligosaccharides having a degree of polymerization (DP) of 2, 3, and 4. The average amount of Neu was determined using a modified resorcinol assay that can measure the amount of neuraminic acid (Neu) and N-acetylneuraminic acid (Neu5Ac) in PSA [19]. After mild base treatment, the oligosaccharides contained ∼25% Neu and there was some degradation of the longer oligosaccharides such that the trimer contained both dimers and trimers and the tetramer contained dimers, trimers and tetramers. The relative ability of the oligosaccharides to inhibit binding of SEAM 3 to the nominal N-Pr MBPS antigen was determined by ELISA. The results are summarized in Table 1. The concentrations of oligosaccharide required to inhibit SEAM 3 binding represent upper limits since the preparations contain a mixture of shorter oligosaccharides in addition to the longest one indicated by DP. Also, the number given for DP does not take into account the possible effect of reducing the C2 carbonyl group of the reducing end residue, which is necessary to prevent degradation during base treatment. Given these considerations, oligosaccharides having a DP of 4 were the best inhibitors of SEAM 3 binding. The ability of the oligosaccharides to inhibit SEAM 3 binding was not affected by incubating them at pH 5 without or with exoneuraminidase at 37°C for 48 hours (data not shown). The treatments enrich the oligosaccharides for chains that have Neu at the non-reducing end [19]. SEAM 3 binding was not inhibited by closely related MCPS (ie. poly α2,9 N-acetylneuraminic acid) either when fully N-acetylated or containing de-N-acetylated residues (Table 1). Taken together, we conclude that SEAM 3 recognizes oligo or polysialic acid derivatives having a DP of 4 or greater, where approximately every fourth residue is Neu, which may be located at the non-reducing end or at internal positions of the polymer (Figure 1).

**Table 1:** Inhibition of SEAM 3 binding by Neu-containing PSA oligosaccharides.

α2,8 PSA derivatives derived from colominic acid		
Average DP	%Neu^a^	IC_50_ b (µg/ml)
>30	<10	5
>30	98	1.7
4	<10	>200
4	25	0.02
3	<10	>200
3	25	1.9
2	<10	>200
2	25	51

α2,9 PSA derivatives derived from MCPS		
Average DP	%Neu^a^	IC_50_ b (µg/ml)
>30	<10	>200
>30	63	>200

a The lower limit of detection is ∼10% Neu in the resorcinol assay.

b Concentration of inhibitor required to inhibit 50% of binding of SEAM 3 to the nominal N-Pr MBPS antigen by ELISA. For comparison, the nominal N-Pr MBPS antigen (DP>30, 16% Neu) used to produce SEAM 3 had an IC_50_ value of 0.01 µg/ml. The concentrations of purified SEAM 3 used in the ELISA were 0.07 µg/ml.

### Immunohistochemical (IHC) staining of normal human skin and primary melanoma tumors

In normal tissues, de-N-acetyl GD3 was reported to be expressed “at low levels in few blood vessels, infiltrating mononuclear cells of the skin and colon and at moderate levels in skin melanocytes” [22]. Higher levels were expressed in melanomas [22]. PSA-NCAM is widely expressed in endodermal, mesodermal, ectodermal and neuro-ectodermal derived tissues at various stages of fetal development, but expression in adults is limited to a few areas of the brain exhibiting neuro-plasticity [29]. To determine whether antigens reactive with SEAM 3 were expressed in normal human skin and primary melanomas, we performed IHC on frozen and formalin-fixed paraffin embedded tissue specimens. The mAb R24 was used to mark GD3. Chammas et al., have reported that formalin treatment of tissue samples can result in modification of de-N-acetyl GD3 amino groups with formaldehyde, resulting in loss of reactivity with anti-de-N-acetyl GD3 mAbs [22]. Also, IHC staining for the presence of GD3 in formalin-fixed paraffin embedded tissues specimens has been described as being sensitive to loss of anti-GD3 reactivity as a result of antigen recovery procedures [30]. We found the respective staining patterns observed for each mAb tested were similar irrespective of which method was used to prepare the specimens, except that staining was decreased to some extent in frozen, unfixed specimens compared to paraffin embedded, formalin-fixed tissues. Also, it was more difficult to obtain large, unbroken sections of tissue in the unfixed specimens. Since fixing the specimens resulted in superior preservation of the tissue structure and greater sensitivity of staining, only the results obtained using fixed tissues are shown.

**Figure 2:**
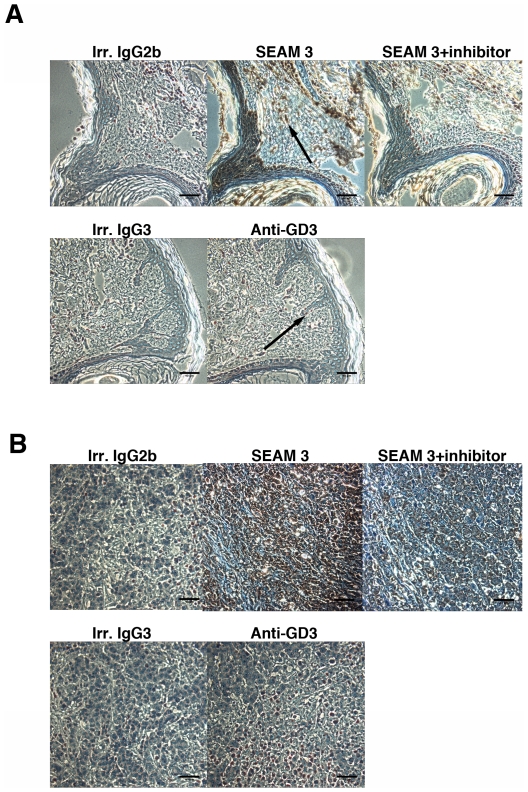
NeuPSA is expressed in normal human skin and in primary melanoma tumors. IHC analysis of SEAM 3 and anti-GD3 binding to normal human skin (A) and a primary melanoma (B). Formalin-fixed normal human skin (A) and primary melanoma (B) were incubated with irrelevant IgG2b, SEAM 3, SEAM 3 with a polysaccharide inhibitor N-Pr MBPS, irrelevant IgG3, and anti-GD3 mAb R24 as indicated. Binding was detected using DAB, which produces a brown precipitate. Counterstaining was performed using hemotoxylin. The arrow in the micrograph of SEAM 3 binding to normal skin shows an example of SEAM 3 marking an infiltrating lymphocyte and for the anti-GD3 micrograph, a melanocyte. Reference bars = 40 µm.

IHC staining of fixed normal human skin shows that SEAM 3 marks cells of the squamous epithelium and infiltrating lymphocytes (Figure 2A), while the negative control irrelevant murine IgG2b and IgG3 mAbs showed no reactivity. In contrast, the anti-GD3 mAb was reactive only with antigens present in a few melanocytes in normal skin (Figure 2A, example indicated by arrow). SEAM 3 staining appeared to be largely cytoplasmic with a distinctive granular appearance. SEAM 3 binding to antigens present in normal skin could be inhibited by greater than 90% when the mAb was pre-incubated with N-Pr MBPS (50 µg/ml). N-Pr MBPS is the polysaccharide antigen used to produce SEAM 3 [18]. The result shows that SEAM 3 binding was mediated by the antibody combining site.

The dark staining of the melanoma specimen resulting from SEAM 3 binding (Figure 2B) shows that S3RA antigens were expressed in the tumor. As with normal skin, SEAM 3 binding to antigens in the melanoma specimen was inhibited with soluble N-Pr MBPS (Figure 2B). The intense staining of the melanoma specimen suggests that S3RA was expressed at higher levels in the tumor compared to normal epithelial cells. All tumor cells within the section, which had dimensions of approximately 1 cm×2 cm, were stained. In contrast, the anti-GD3 mAb exhibited heterogeneous binding to the tumor tissue with some cells marked by the mAb while others showed no staining (Figure 2B).

In addition to the example primary human melanoma shown in Figure 2B, an array of 12 smaller primary human melanoma specimens taken from skin, esophageal, parotid, and rectal tumors were evaluated for SEAM 3 and irrelevant IgG2b reactivity by IHC. All the melanoma specimens in the array were reactive with SEAM 3 while the irrelevant IgG2b mAb showed no reactivity (data not shown). The intensity of staining resulting from SEAM 3 binding in the melanoma tumor array was similar to the example shown in Figure 2B. Samples of normal skin taken from areas adjacent to some of the melanomas that were included in the specimen array exhibited the same pattern of SEAM 3 reactivity as that shown in Figure 2A with normal skin (data not shown).

### Expression of S3RA in cancer cell lines

Since the de-N-acetyl sialic acid-containing derivative of the disialyloganglioside GD3 has been reported to be present in melanoma cell lines [24] as well as in primary human tumors [26] and we found that SEAM 3 was reactive with primary melanomas (Figure 2B), we performed flow cytometry to measure SEAM 3 binding with four human cancer cell lines that express different Sia-containing antigens (Table 2).

**Table 2:** Expression of PSA-NCAM and GD3 in human cancer cell lines tested.

Sia antigen	Cell line			
	SK-MEL-28	Jurkat	CHP-134	SH-SY5Y
PSA-NCAM	−	−a	+	+ [42]
GD3	+ [43]	−	+	−

a Recent studies have suggested that Jurkat cells may transiently express GD3 during Fas (CD95)-signaling [44] and normal human leukocytes have been shown to express PSA-NCAM [10].

**Figure 3:**
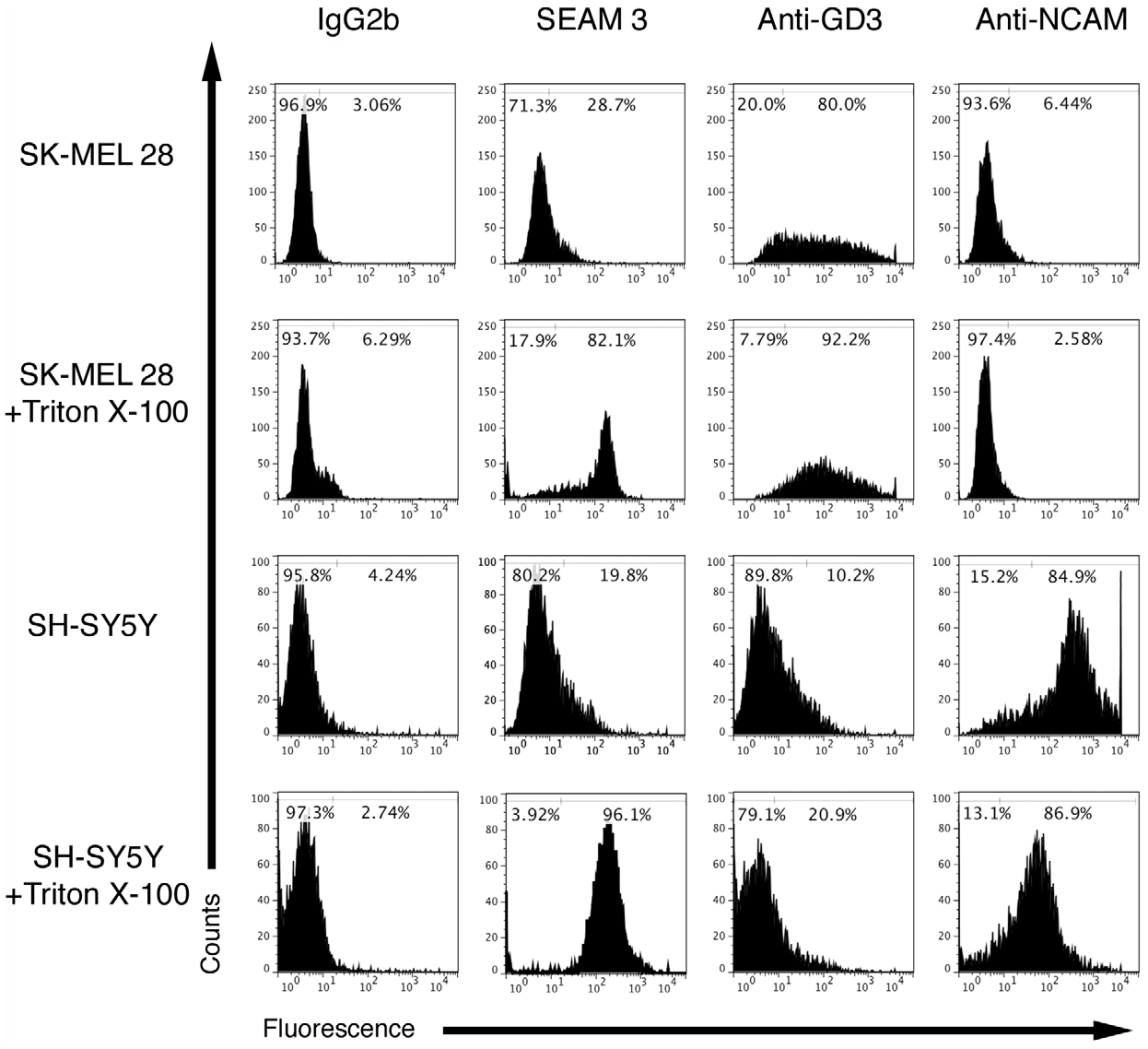
Intracellular localization of de-N-acetyl PSA in melanoma and neuroblastoma cell lines. SK-MEL-28 and SH-SY5Y cells were either untreated to detect surface binding (upper panel in each set of two panels), or treated with Triton-X 100 to detect intracellular binding (lower panel) by flow cytometry. Cells were incubated with 5 µg/ml of each primary antibody, followed by incubation with 2 µg/ml Alexa Fluor 488-conjugated secondary antibody. Irrelevant murine IgG2b, IgG3, and IgG1 mAbs were used as negative controls for SEAM 3, anti-GD3, and anti-NCAM, respectively, and were used to determine baseline fluorescence. Binding was detected using Guava EasyCyte flow cytometer. Gates used to define cells positive for binding are indicated at the top of each histogram.

Figure 3 shows binding of the subclass-matched irrelevant mAb IgG2b compared to SEAM 3, the anti-GD3 mAb R24 and anti-NCAM (CD56) mAb binding to SK-MEL-28 melanoma and SH-SY5Y neuroblastoma cells in the absence and presence of Triton X-100 treatment, as indicated. Gates defining cells as positive for binding were set using subclass matched isotype controls and are indicated on each histogram shown in Figure 3. In the absence of detergent, 29% of SK-MEL-28 cells were positive for SEAM 3 binding compared to 80% for anti-GD3 and 3% for the irrelevant mAb. However, when the cells were made permeable to the mAbs by detergent treatment, 82% of the SK-MEL-28 cells were positive for SEAM 3 binding and the relative fluorescence increased more than 10-fold. The percent of anti-GD3 positive cells increased slightly to 92%. Binding of the anti-NCAM mAb was comparable to the irrelevant IgG1 mAb in the presence or absence of detergent and, therefore, N-CAM was not expressed in SK-MEL-28 cells. For the SH-SY5Y cell line, 20% of the non-detergent treated cells were positive for SEAM 3 binding, which increased to 96% in the presence of detergent (Figure 3). Also, the relative fluorescence increased by more than 10-fold. Greater than 85% of SH-SY5Y cells were positive for anti-NCAM and less than 21% for anti-GD3 binding in both intact or permeabilized cells. The results summarized in Table 3 for the four cell lines show that the percent of cells positive for SEAM 3 binding and the mean fluorescence of the cells was variable among the cell lines tested. However, the intact cells of each cell line were largely negative for SEAM 3 binding. In contrast, nearly all cells of each cell line contained intracellular SEAM 3-reactive antigens (S3RA). The predominant intracellular distribution of S3RAs did not correspond to the distribution of GD3 or PSA-NCAM, which were mainly localized on the cell surface. The result suggests that S3RAs were not de-N-acetyl Sia derivatives of either GD3 or PSA-NCAM or that the intracellular counterparts of these molecules were not reactive with the respective mAbs.

**Table 3:** Summary of SEAM 3 binding to cancer cell lines determined by flow cytometry.

Cell line	Percent positive (geometric mean fluorescence)			
Jurkat	IgG2b	SEAM 3	Anti-GD3	Anti-NCAM
−Triton X-100	5 (3)	16 (5)	8 (3)	6 (3)
+Triton X-100	3 (3)	95 (74)	57 (12)	1 (2)
SK-MEL-28				
−Triton X-100	3 (4)	29 (8)	80 (73)	6 (5)
+Triton X-100	6 (5)	82 (65)	92 (113)	5 (4)
CHP-134				
−Triton X-100	5 (5)	25 (15)	64 (48)	85 (169)
+Triton X-100	4 (3)	93 (118)	91 (92)	83 (29)
SH-SY5Y				
−Triton X-100	4 (4)	20 (9)	10 (8)	85 (238)
+Triton X-100	3 (3)	96 (148)	21 (4)	87 (39)

To demonstrate specificity of binding, fixed and permabolized Jurkat or SK-MEL-28 cells were incubated with SEAM 3 in the absence and presence of the N-Pr MBPS inhibitor. Unlike the fixed tissue specimens however, the relatively high concentrations of SEAM 3 (5 µg/ml) in combination with the soluble N-Pr MBPS inhibitor produced ambiguous results. In some experiments partial inhibition was observed but in others the polysaccharide inhibitor had either no effect or resulted in an increase in binding. It is possible that the variable effects of adding N-Pr MBPS to the binding reaction resulted from the propensity of NeuPSA derivatives present in the N-Pr MBPS preparation to form aggregates that are too large to escape the permeablized cells or that the cells have receptors for NeuPSA that also bind to N-Pr MBPS.

#### Fluorescence microscopy

**Figure 4:**
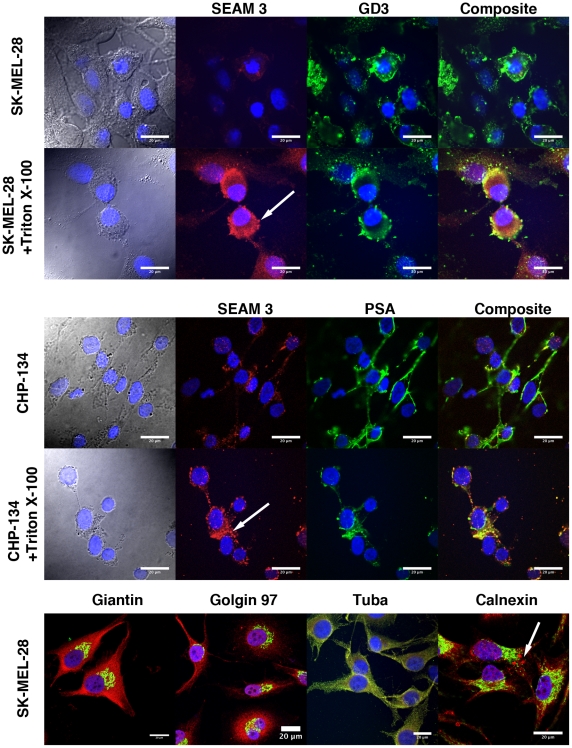
Immuno-fluorescence analysis of SEAM 3 binding to human SK-MEL-28 melanoma and CHP-134 neuroblastoma cells. Light micrographs of SK-MEL-28 and CHP-134 show the general shape of cells with nuclear DNA indicated in blue. Anti-NeuPSA mAb SEAM 3 binding (in red) to SK-MEL-28 and CHP-134 cells not treated or treated with Triton X-100 to permeablize the cells, was compared to anti-GD3 and –PSA in green, as indicated. Subcellular localization of S3RA in SK-MEL-28 cells with Golgi (giantin, golgin 97 and Tuba) and ER (calnexin) markers are shown in the bottom panel in yellow. Arrows indicate granular vesicular-like structures with relatively intense SEAM 3 staining. Reference bars = 20 µm.

Immuno-fluorescence microscopy (IFM) was used to further investigate the cellular location of SEAM 3-reactive epitopes in detergent untreated and treated SK-MEL-28 and CHP-134 cells (Figure 4). The results of flow cytometry binding experiments with SK-MEL-28 cells not treated or treated with detergent (Figure 3) suggested that SEAM 3 reactive epitopes had a cellular localization that was different from GD3 and PSA-NCAM. Consistent with these results, only a subset of SK-MEL-28 and CHP-134 cells not treated with detergent were reactive with SEAM 3 by IFM (red fluorescence in Figure 4). As shown in Figure 4, SK-MEL-28 cells that were most reactive with SEAM 3 were “rounded up” cells with relatively condensed nuclear DNA as shown by DAPI staining. Elongated cells were less reactive with SEAM 3 (compare SK-MEL-28 cells in light micrograph shown in Figure 4 with IFM). In contrast, anti-GD3 labeled all cells (green fluorescence as indicated in Figure 4). However, when the SK-MEL-28 cells were treated with detergent, all cells were positive for SEAM 3 binding (Figure 4). In the detergent-treated cells, SEAM 3 reactivity was dispersed throughout the cytoplasm in granule-like structures (Figure 4, example indicated by an arrow). Anti-GD3 reactivity was characterized by a diffuse pattern of staining with spots of concentrated reactivity in both untreated and detergent-treated cells (Figure 4). There was some degree of co-localization evident in composite images of SEAM 3 and anti-GD3-labled cells (yellow in Figure 4) but clear differences as well. Thus, SEAM 3 reactivity was not directly correlated with GD3 other than incidental localization to the membrane in the absence or presence of detergent and expression of S3RAs was limited to a subset of cells.

In CHP-134 cells, SEAM 3-reactive epitopes (red fluorescence in Figure 4 CHP-134 cells as indicated) were mainly localized to borders of contact between cells. PSA antigens, as detected with the anti-PSA mAb 2-1-B [31] (green fluorescence in Figure 4 as indicated), were also located mainly in regions of cell-cell contact. Although the fluorescence resulting from SEAM 3 binding was lower, both the relative intensity and distribution of fluorescence resulting from anti-PSA binding was similar to that of SEAM 3 in intact cells as shown by the yellow color resulting from superposition of red and green fluorescence in the composite image (Figure 4). This was indicative of the SEAM 3-labeled antigen being located on the cell surface. Moreover, SEAM 3 labeling was correlated with anti-PSA labeling (Manders' coefficient for green = 0.998) and to a lesser extent with anti-NCAM labeling (Manders' coefficient for green = 0.642; micrographs not shown) in either the absence or presence of Triton X-100. However, when the cells were treated with detergent there was less overlap between SEAM 3 and anti-PSA labeling (indicated by relatively larger amount of red fluorescence in the detergent treated composite micrograph) suggesting that there was a fraction of antigens recognized by the mAbs in common and distinct antigens recognized separately.

Since in SK-MEL-28 cells the majority of antigens reactive with SEAM 3 were located in granular like structures inside cells, we performed IFM using markers for the Golgi and endoplasmic reticulum (ER) to determine whether SEAM 3 reactivity was associated with those particular subcellular organelles. Golgi markers included giantin and golgin-97 for cis/medial and trans Golgi membranes, respectively, and Tuba, a multifunctional protein thought to be involved in regulating cell junction and endocytic trafficking [32] of Golgi-derived vesicles in the cytoplasm [33]. Calnexin, a molecular chaperone protein was used as an ER marker [34]. As shown by the presence or lack of yellow fluorescence in composite images, SEAM 3 was substantially co-localized with the Golgi and ER (as indicated in Figure 4) markers. Co-localization was analyzed further using JACoP [35] in Image J. All of the Golgi and ER markers were found to have high colocalization for the marker versus SEAM 3 (Manders' coefficients for green all greater than 0.92) with obvious distribution of S3RAs outside of the organelles as well. The closest overall colocalization was between SEAM 3 and Tuba as shown in Figure 4. Thus, NeuPSA antigens detected using SEAM 3 were mainly located inside cells and associated with the Golgi, ER and cellular matrix, but were also present on the surface of subpopulations of each cell line.

### Expression of polysialyltransferases PST and STX in cancer cell lines

**Figure 5:**
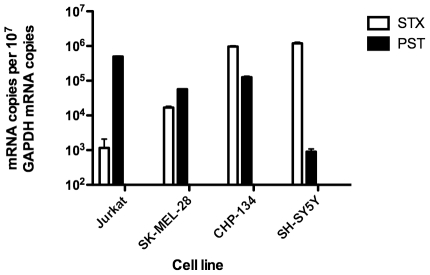
Absolute quantification of STX and PST mRNA of cancer cell lines by qRT-PCR. 1 µg of total RNA from each cell line was reverse-transcribed into cDNA, then used as the template in the qRT-PCR reaction. The mRNA copy number of STX, PST, and GAPDH determined from a standard curve of serially dilute standards is shown in the graph as target mRNA copies per 10^7^ GAPDH mRNA copies. Error bars are the SEM of triplicate measurements from three independent populations of each cell line.

The neuroblastoma cell lines CHP-134 and SH-SY5Y were known to express high levels of PSA in the form of PSA-NCAM, while Jurkat and SK-MEL-28 cells were not known to express PSA or NCAM. Since NeuPSA is likely to be derived from PSA, we measured the expression of the α2,8 polysialyltransferases ST8Sia2 (STX) and ST8Sia4 (PST) mRNA in the four cell lines by quantitative PCR. SH-SY5Y cells have been shown to express both STX and PST [36], and were used for comparison of STX and PST expression in the other cell lines. Using absolute quantification, we determined STX expression was highest in SH-SY5Y cells, with 1.2×10^6^ copies/1×10^7^ copies of GAPDH (Figure 5). CHP-134 expressed similar levels of STX, while SK-MEL-28 cells expressed nearly 100-fold less and Jurkat cells expressed nearly 1000-fold less STX. SH-SY5Y cells expressed the least amount of PST compared to the other three cells lines, with 9.0×10^2^ copies/1×10^7^ copies GAPDH. CHP-134, SK-MEL-28, and Jurkat cells expressed 60, 140, or 550-fold more PST, respectively, compared to SH-SY5Y cells (Figure 5).

### Dependence of SEAM 3 reactivity with SK-MEL-28 cells on the expression of polysialyltransferase PST

**Figure 6:**
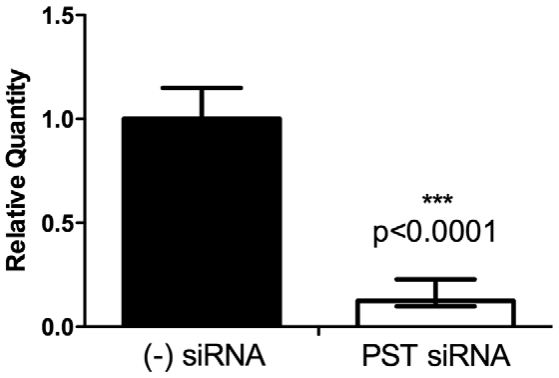
Relative quantification of PST mRNA in SK-MEL-28 cells treated with scrambled siRNA or PST-specific siRNA. SK-MEL-28 cells were transiently transfected with 50 nM siRNA for 72 hours in triplicate, then total RNA from each cell line was reverse-transcribed into cDNA and used as the template in the qRT-PCR reaction. Relative quantification was determined using the comparative CT method, normalized to GAPDH mRNA.

To establish that SEAM 3 is reactive with an antigen that is derived from PSA, we investigated whether inhibiting the expression of polysialyltransferase PST mRNA in SK-MEL-28 cells with PST-specific small interfering RNA (siRNA) would result in a decrease in SEAM 3-positive cells by flow cytometry. Negative control scrambled siRNA or PST specific-siRNA was transfected into SK-MEL-28 cells and after 72 hours, the relative quantity (RQ) of PST present was significantly decreased (P<0.0001) in the cells transfected with PST siRNA (RQ = 0.124) compared to cells transfected with negative control siRNA (RQ = 1) (Figure 6).

In this experiment, only the subset of cells transfected with the siRNA and depleted of pre-existing PSA derivatives would be expected to show deceased reactivity with SEAM 3 if the antigen reactive with the mAb was dependent on the activity of PST. Despite these limitations, in three separate experiments with two performed in triplicate, the fraction of SEAM 3-positive cells was significantly decreased in cells transfected with PST siRNA compared to cells transfected with scrambled siRNA (Table 4). The result clearly shows that antigens recognized by SEAM 3 in SK-MEL-28 cells were dependent on the expression of PST.

**Table 4:** Effect of polysialyltransferase PST siRNA on SEAM 3 binding to SK-MEL-28 cells.

Experiment (replicates)	Percentage of SEAM 3-positive cells±SD^a^		P value^b^
	Scrambled PST siRNA	PST siRNA	
1 (1)	80	42	NA
2 (3)	82±0.6	74±2	0.02
3 (3)	82±0.4	59±0.7	<0.0001

a Binding to Triton X-100-treated SK-MEL-28 cells 72 hours after transfection with the indicated siRNA was measured by flow cytometry as shown in Figure 3.

b P values comparing the mean percentages of SEAM 3-positive cells were determined using an unpaired two tailed t test.

### Cytotoxic functional activity of SEAM 3 against cancer cells

**Figure 7:**
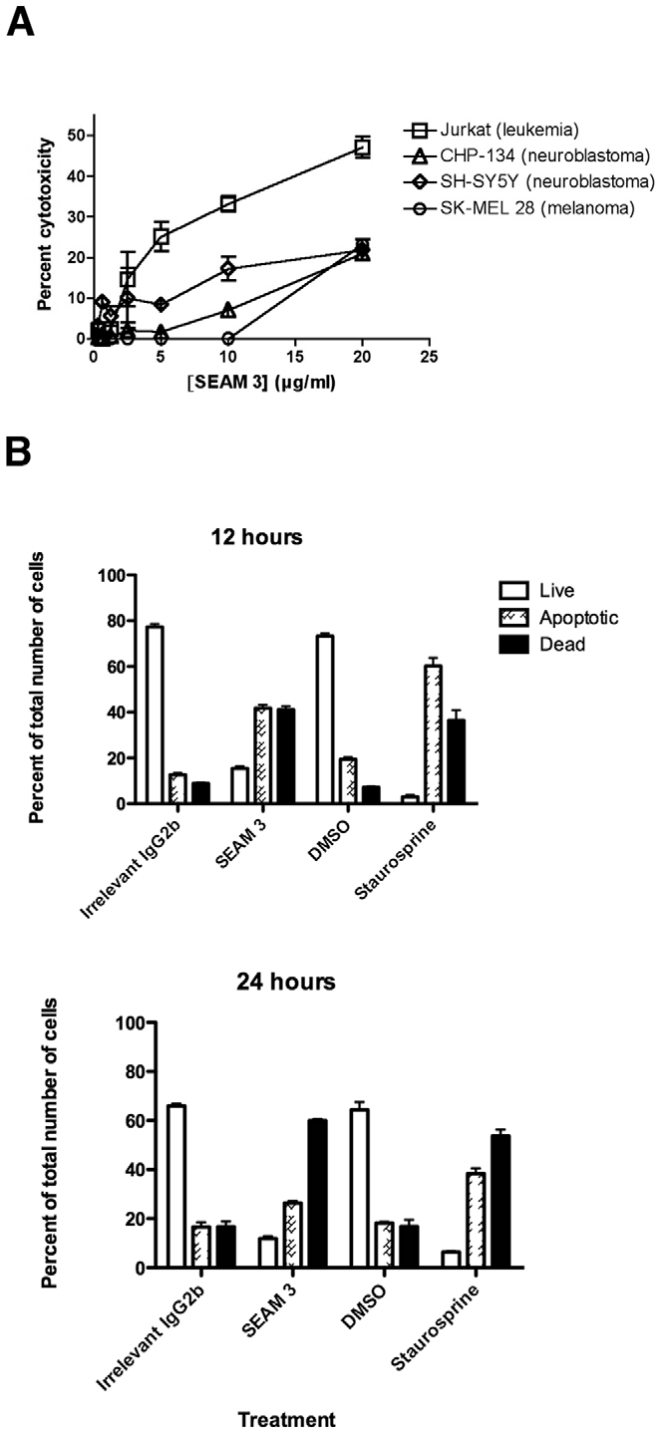
SEAM 3 mediates antibody dependent cytotoxicity by inducting apoptosis. (A), Antibody dependent cytotoxicity of SEAM 3 against SK-MEL-28, CHP-134, SH-SY5Y, and Jurkat cells as measured by LDH release assay. Each cell line was incubated with increasing concentrations of SEAM 3 for 16 hrs. LDH release was measured and percent cytotoxicity was determined using spontaneous release and maximal release following treatment with Triton X-100. (B), Analysis of SEAM 3 mediated apoptosis against SK-MEL-28 melanoma cells by flow cytometry. SK-MEL-28 cells were incubated with an irrelevant IgG2b mAb (5 µg/ml), DMSO, 0.1 µM Staurosporine, or 5 µg/ml SEAM 3 for 12 or 24 hours. Cells were then stained with fluorescently labeled annexin V and propidium iodide and the fraction of live (open bars), apoptotic (cross-hatched bars), and dead cells (black bars) was measured by flow cytometry.

The function of S3RA in cells is unknown. To determine what effect interfering with the function of S3RA expressed on the cell surface might have, cells were incubated overnight with SEAM 3 at mAb concentrations used in the binding studies described above. Under microscopic examination, the cells exhibited characteristics of undergoing apoptosis (data not shown). Based on this initial observation, we used two independent assays to measure the effect of SEAM 3 on the viability of the four cell lines: flow cytometry with the ViaCount reagent and a lactate dehydrogenase (LDH) release assay. The first is based on membrane permeable and impermeable DNA-binding fluorescent dyes and the LDH assay on the release of LDH resulting from the loss of membrane integrity. The results for the concentration-dependent effect of SEAM 3 on the viability of the four cell lines after 16 hours of incubation using the LDH assay are shown in Figure 7A. Purified SEAM 3 was cytotoxic against the four cell lines tested but the effect was variable for each cell line. Jurkat cells were the most sensitive to SEAM 3-mediated antibody dependent cytotoxicity (ADC) while SK-MEL-28 cells were the least sensitive. Approximately 45% of Jurkat cells and 25% of SK-MEL-28, CHP-134, and SH-SY5Y cells were killed at the highest SEAM 3 concentration tested. SEAM 3-mediated cytotoxicity appeared to level off at a fixed fraction of cells. For example, ADC against Jurkat and SH-SY5Y cells mediated by SEAM 3 approached a maximum at about 10 µg/ml of SEAM 3 with only a marginal increase in killing at 20 µg/ml (Figure 7A). The results may reflect the fact that both the number of cells and the amount of S3RA expressed by the cells was variable among the cell lines (see Table 3) and that the amount of antigen remains relatively constant in the population of cells over the 16 hr time period. Results similar to those shown in Figure 7A, were obtained using the Guava ViaCount assay (data not shown). To demonstrate specificity of SEAM 3 ADC activity, we attempted to inhibit killing with soluble N-Pr MBPS. However, the inhibition experiment was confounded because soluble N-Pr MBPS was taken up by live cells resulting in a marked increase in the expression of S3RA (B. T. Hagen and G. R. Moe, unpublished).

### SEAM 3 binding induces apoptosis in Jurkat and SK-MEL-28 cells

To determine whether SEAM 3 cytotoxicity resulted from apoptosis of Jurkat and SK-MEL-28 cells, we compared the effect of SEAM 3 binding on the cells to the effect of staurosporine, a general kinase inhibitor that is a classic inducer of apoptosis [37]. In the example shown in Figure 7B, SK-MEL 28 cells were incubated for 12 and 24 hours with the irrelevant mAb, DMSO, staurosporine, or SEAM 3, and the number of live, apoptotic and dead cells was quantified using a flow cytometric assay that measured the appearance of phosphatidylserine on the cell surface through annexin V binding and DNA content by propidium iodide staining. As shown in Figure 7B, treatment with SEAM 3 or staurosporine increased the percent of apoptotic and dead cells after 12 hrs and 24 hrs compared to the IgG2b and DMSO controls, respectively. The results for Jurkat cells incubated with 10 µg/ml SEAM 3 were similar to those shown for SK-MEL 28 cells in Figure 7B (data not shown).

## Discussion

Little is known about the expression of de-N-acetyl sialic acid-containing antigens in normal or diseased human tissues. Studies published to date have presented evidence for the expression of gangliosides GM3 and GD3 containing Neu in some cancer cell lines and Neu-containing GD3 in primary human melanoma tumors [23], [24], [26]. Our laboratory has shown that NmB strains and the protozoan parasite *Leishmania major* express NeuPSA [19], [20], [38], [39], which had not been described previously for any organism. In this study, we have shown by IHC that the anti-NeuOSA/PSA mAb, SEAM 3, reacts with antigens that are expressed in normal squamous cells, melanocytes and in primary human melanomas. The S3RA appeared to be produced at considerably higher levels in primary melanomas compared to normal melanocytes, suggesting the S3RA expression is abnormal in the tumor cells. Unlike gangliosides and known polysialylated surface proteins, such as PSA-NCAM, S3RA appeared to be located mainly inside cells. In addition to primary tumors, S3RA was detected in melanoma, leukemia and neuroblastoma cell lines. Although nearly all cells in each cell line were positive for SEAM 3 binding when the cells were made permeable to the mAb by treatment with detergent, only a subpopulation of cells ranging from 16% to 29% of intact cells expressed S3RA on the cell surface. The subset of SK-MEL-28 melanoma cells that were most reactive with SEAM 3 were rounded up and had condensed nuclei, characteristic of dividing cells.

The majority of S3RA being located inside cells is unusual compared to known polysialylated proteins. As far as we are aware, the only polysialylated antigens that are located mainly inside cells, associated with the Golgi and ER, but can also be found on the cell surface are the α2,8 polysialyltransferases PST and STX [8]. We found that both PST and STX mRNAs were expressed at variable levels in the four tumor cell lines tested. In addition, we found that transfecting SK-MEL-28 cells with PST-specific siRNA decreased the number of cells that expressed S3RAs. The result directly links SEAM 3 binding to the PST polysialyltransferase. However, S3RA was also found outside of organelles normally associated with polysialylated antigens, which raises questions about whether S3RA is in fact a NeuOSA/PSA derivative or an undefined epitope that depends on the expression of PST.

While the identity and biological function of S3RA is not known, SEAM 3 binding to S3RA resulted in antibody dependent cytotoxic activity against the four cell lines tested by inducing apoptosis. The ability of SEAM 3 to effect intracellular changes by binding to antigens on the cell surface suggests that S3RA may have some role in signal transduction. For example, NCAM is known to modulate the activity of several receptor proteins and modification of NCAM with PSA in turn modulates the activity of NCAM [9].

The effects of SEAM 3 binding on the tumor cell lines described here raise the intriguing possibility that antibodies like SEAM 3 that are elicited by NeuSia-based vaccines may be useful for the detection, prevention or treatment of cancers that overexpress S3RA or related antigens. Furthermore, it may be possible to effect changes in the production of S3RA with inhibitors of PST, STX or, possibly, the putative PSA de-N-acetylases that may be needed to produce S3RA.

## Materials and Methods

### Materials

Cell culture medium along with all supplements were purchased from UCSF Tissue Culture Facility (San Francisco, CA) except fetal bovine serum (Gemini Bioproducts, West Sacramento, CA). Anti-GD3 mAb R24 was purchased from Covance, Dedham, MA. Anti-CD56 and anti-CD95 mAbs were from BioLegend, San Diego, CA. Anti-golgin 97 mAb was from Invitrogen, Carlsbad, California. Anti-giantin, anti-Tuba, and anti-calnexin were from Abcam, Cambridge, MA. Irrelevant control subclass mAbs were from Southern Biotech, Inc. Birmingham, AL. All anti-mouse secondary antibodies conjugated with Alexa Fluor fluorochromes were obtained from Invitrogen. Guava Viacount Reagent was purchased from Millipore, Billerica, MA. The LDH release assay was from Promega, Madison, WS. Vybrant Apoptosis Assay Kit #3 was purchased from Invitrogen.

### PSA derivatives

MBPS obtained as colominic acid was from Sigma-Aldrich. *Neisseria meningitidis* group C capsular polysaccharide (poly α2,9 N-acetylneuraminic acid) was a gift from Jo Anne Welsch at CHORI. N-propionyl MBPS (N-Pr MBPS), was prepared as described previously [19]. Partially de-N-acetylated oligosaccharide derivatives were prepared from purified MBPS dimers, trimers and tetramers (EY Laboratories, Inc., San Mateo, CA) by combining the oligosaccharides with sodium borohydride in water at a ratio of 10∶1 (weight/weight) and incubating the reaction mixture at ambient temperature for 18 hrs. After extensive dialysis in water (1 kDa cut-off membrane), the solutions were lyophilized. The oligosaccharides were analyzed by high performance anion exchange chromatography (Dionex, Sunnyvale, CA) with pulsed ampermetric detection (HPAC-PAD) on a CarboPac 10 column (Dionex) in 0.1 M NaOH with a 0.1 M to 1 M gradient of NaOAc. De-N-acetyl GD3 and GM3 were prepared and characterized by high performance thin layer chromatography (HPTLC) as described by Sonnenburg et al. [40]. The amount of neuraminic acid (Neu) and N-acetylneuraminic acid (Neu5Ac) was determined using a modified resorcinol assay described in [19]. Subsequently, we have determined that the assay is best performed in sealed glass hydrolysis tubes (Pierce, Rockford, IL) in a boiling water bath for 7 minutes for Neu and, separately, 30 minutes for Neu5Ac. The Neu concentration was measured from the absorbance at 500 nm of the colored complex in the aqueous layer, which was compared to a standard curve generated using several concentrations of fully de-N-acetylated colominic acid. The results obtained using this method on partially de-N-acetylated colominic acid containing 16%, 23%, 41%, 50%, 57%, and 79% Neu were nearly the same as those measured on the same samples by NMR (data not shown). Neu5Ac was determined from the absorbance at 580 nm of the alcohol layer and compared to a standard curve based on unmodified colominic acid.

### Inhibition ELISA

Inhibition of SEAM 3 binding to a solid phase dodecylamine derivative of N-Pr MBPS was performed as previously described [18], [19].

### Tissue Culture

SK-MEL-28 human melanoma cells were purchased from ATCC (HTB-72). Cells were grown routinely in RPMI 1640 medium containing 0.1 mM non-essential amino acids, 1.0 mM sodium pyruvate, 2 mM glutamine, penicillin/streptomycin and 10% fetal bovine serum (FBS) at 37°C in 5% CO_2_. Confluent cells were sub-cultured (1∶3 to 1∶8) by treating with 0.25% (w/v) Trypsin/0.53 mM EDTA solution, triturating to release adherent cells and washing in media before re-seeding into new growth medium. SK-MEL-28 cells were only used up to passage 10 from the original ATCC stock of cells. Jurkat cell clone E6-1, a human acute lymphoblastic leukemia T cell line was purchased from ATCC (TIB-152). Jurkat cells were grown in the same media as SK-MEL-28 cells in 5% CO_2_ at 37°C and subcultured every 3 days with a split ratio of about 1∶5. CHP-134 and SH-SY5Y, human neuroblastoma cell lines were originally purchased from the European Collection of Cell Cultures (ECACC) and ATCC (CRL-2266), respectively. CHP-134 cells were routinely grown in RPMI 1640 medium containing 0.1 mM non-essential amino acids, 1.0 mM sodium pyruvate, 2 mM glutamine, penicillin/streptomycin and 10% FBS at 37°C in 5% CO_2_. Confluent cells were sub-cultured (1∶3 to 1∶5). The cells were released from the plate with repeated pipetting then re-seeded into new growth medium. SH-SY5Y cells were routinely grown in DMEM/F12 50% mix medium containing penicillin/ streptomycin and 10% FBS at 37°C in 5% CO_2_. Confluent cells were sub-cultured (1∶3 to 1∶5) by treating with 0.25% (w/v) Trypsin/0.53 mM EDTA solution, triturating to release adherent cells and washing in media before re-seeding into new growth medium.

### Immunohistochemistry

Unfixed frozen and formalin fixed paraffin-embedded samples were either prepared locally from tissues obtained under an IRB approved protocol from the National Disease Research Interchange (normal skin and primary human melanoma) or purchased from USBiomax (human melanoma arrays). Formalin fixed paraffin-embedded tissues were deparaffinized in xylene, and then rehydrated in a series of 95%, 90%, 75%, and 50% alcohol incubations. Antigen retrieval was conducted using a Decloaking Chamber (BioCare Medical) and Reveal Decloaker (BioCare). Unfixed or frozen sections were stained using a Nemesis 3600 (BioCare) automated stainer and IntelliPATH FLX Universal HRP Detection Kit (BioCare). Samples were blocked for 5 minutes with peroxidase block, incubated for 1 hour with 0.5 µg/ml IgG2b, SEAM 3, IgG3, or anti-GD3 mAb R24 primary antibody, 20 minutes with secondary, 20 minutes with tertiary antibody, 5 minutes with 3, 3′ diaminobenzidine (DAB), and finally counterstained with hemotoxylin. All slides were analyzed using a Zeiss Axioplan 2 Upright Light/Fluorescence Microscope with digital video capture. Digital images were obtained using Q Capture (Technical Instruments, San Jose, CA).

### mAb Binding Assay

Adherent cells were detached from the flask with either 0.25% (w/v) Trypsin/0.53 mM EDTA (SK-MEL-28 and SH-SY5Y) or pipetting (CHP-134) before being collected into a 96-round bottom well plate. Non-adherent Jurkat cells were directly collected into wells of a 96-round bottom well plate. Cells were counted and viability was determined using Guava Viacount Reagent and Guava EasyCyte capillary flow cytometer (Millipore). Cells used consistently had greater than 90% viability. Approximately 10^5^ cells per well were spun at 500×g for 5 minutes and washed with ice cold Dulbecco's phosphate buffered saline (D-PBS) without Mg^2+^ or Ca^2+^ salts (CMF D-PBS). Cell surface binding was determined using live cells. Intracellular binding was determined using fixed, Triton X-100 treated cells. Cells were fixed with ice-cold 0.37% (v/v) formaldehyde in PBS for 15 minutes. Cells were pelleted by centrifugation as above and incubated in 0.05% (w/v) Triton X-100 for 5 minutes. Live or fixed cells were incubated with 5 µg/ml primary antibody diluted in 3% goat serum for 1 hour at room temperature. Cells were washed by pelleting and resuspending three times with ice-cold CMF D-PBS, and then secondary goat anti-mouse antibody conjugated to a specific fluorochrome was applied for at least 30 min at 4°C in the dark. The cells were washed three additional times then binding was analyzed using the Guava EasyCyte flow cytometer (Millipore). Negative control samples were treated with a subclass-matched irrelevant antibody, which were used to determine baseline fluorescence. Positive controls for each cell type were anti-CD3 for Jurkat cells, anti-GD3 (R24) for SK-MEL-28 cells, and anti-NCAM (CD56) for CHP-134 and SH-SY5Y cells. FlowJo (TreeStar) was used for data analysis.

### Laser scanning confocal microscopy

The preparation of slides and acquisition of images was performed by C. Paul Plested from CHORI or by L.M.S. CHP-134 or SK-MEL-28 cells (∼10^5^) were cultured on multi-well microscope slides that had been treated with poly-L-lysine (Nunc, ThermoFisher Scientific). After an overnight incubation cells were gently washed with PBS and fixed with ice-cold 1% (v/v) formaldehyde in PBS. After 20 minutes, the cells were washed with PBS before being blocked in a solution of 3% (v/v) goat serum without or with 0.05% (w/v) Triton X-100 for 1 hour. The primary antibodies were added and incubated for 2 hours at ambient temperature or overnight at 4°C. The primary antibodies included anti-NeuPSA mAb SEAM 3 [18], anti-PSA 2-1-B [31], anti-CD56 (Sigma), anti-GD3 and R24 (Covance). Cells were gently washed as described above with ice-cold PBS before goat anti-mouse isotype-specific secondary antibodies conjugated to Alexa Fluor 488, Alexa Fluor 546, or Alexa Fluor 633 were applied for at least 1 hour at 4°C in the dark. After another series of gentle washes, a hardening mounting medium containing DAPI was applied (VECTASHIELD, Vector Labs).

Subcellular localization of SEAM 3 to the ER was determined using anti-calnexin (Abcam) and to the Golgi using anti-giantin (Abcam), anti-golgin 97 (Invitrogen) and anti-Tuba (Abcam).

SK-MEL-28 cells were grown on coverslips overnight in their normal growth media. The next day, cells were briefly rinsed with PBS, then fixed with 4% paraformaldehyde for 15 minutes at room temperature. Cells were washed twice for 5 minutes with PBS, treated with 0.2% Triton X-100 for 10 minutes, then washed with PBS three times for 5 minutes. Next, cells were blocked in 1% BSA/PBST containing 0.3 M glycine for 30 minutes, followed by incubation with primary antibody in 1% BSA/PBST. After one hour, cells were washed three times with PBS, then incubated for 30 minutes with secondary antibody in 1% BSA. Cells were washed three times with PBS. Nuclei were stained using 0.5 ug/ml Hoechst 33258 (Invitrogen) for 1 minute, rinsed with PBS, then the coverslips were mounted to slides using Prolong Antifade (Invitrogen). Confocal images were obtained using a Zeiss Meta510 (The Biological Imaging Facility, University of California, Berkeley, CA) or Zeiss LSM710 (CHORI) laser scanning confocal microscopes and were analyzed using ImageJ Software [41] and colocalization with JACoP [35]. Control antibodies and secondary antibodies applied alone were routinely used to assess background fluorescence.

### Quantitative Real-Time PCR

RNA was isolated from SH-SY5Y, CHP-134, SK-MEL-28 and Jurkat cells using an RNeasy Mini Kit (Qiagen). Cells were lysed using a 20-gauge needle. RNA purity and quantity was analyzed using a Thermo Scientific Nanodrop spectrophotometer. One µg of RNA was reverse transcribed using Qiagen's Omniscript RT Kit to synthesize cDNA. 2 µl of cDNA was mixed with Taqman Gene Expression Master Mix, and the appropriate pre-designed Taqman primer/probe mixture (Invitrogen). The cDNA was amplified using an ABI 7500 Fast Real Time PCR System (Applied Biosystems). Absolute quantification of GAPDH, STX, and PST mRNA for each sample was determined using standard curves generated using serially diluted standards containing the same sequence as the amplicon. Absolute quantity is expressed as copies of target gene/10^7^ copies GAPDH.

### siRNA transfection and analysis

SK-MEL-28 cells were grown to approximately 80% confluency overnight. siRNA was mixed with Lipofectamine RNAiMAX (Invitrogen) and prepared according to the manufacturer's instructions. 50 nM of either Silencer Select negative control #1 (Invitrogen) or Silencer Select ST8Sia4 (Invitrogen) was reverse transfected into the cells and incubated for 72 hours at 37°C. After 72 hours, gene knock-down was determined by real-time qPCR. RNA was processed and cDNA was amplified as above, then relative quantity was determined by comparing to GAPDH mRNA. NeuPSA expression was determined using flow cytometry by comparing SEAM 3 binding as described above in the negative control to the PST knock-down cells.

### Cell Viability/Cytotoxicity Studies

Cells (∼10^5^ per well) were plated onto a flat bottom 96-well tissue culture plate and incubated in their normal growth media with IgG2b irrelevant isotype control, SEAM 3, anti-CD95, anti-GD3, or staurosporine at 37°C for 16 or 24 hours. Cell viability was determined after 24 hours using ViaCount Reagent, as per manufacturer's instructions. Briefly, adherent cells were released from the tissue culture plate as described above. Cells in suspension and adherent cells were collected by centrifugation and resuspended in ViaCount Reagent. The viability was analyzed using a pre-set program on the Guava EasyCyte flow cytometer. Cytotoxicity was, alternatively, determined using CytoTox 96 Non-Radioactive Cytotoxicity Assay (Promega). In this assay cells were incubated with media to determine spontaneous LDH release, Lysis Solution to determine maximum LDH release, or SEAM 3 for 16 hours at 37°C, then the supernatant was collected and incubated with Substrate Mix for 30 minutes, the reaction was stopped using Stop Solution, and the absorbance at 490 nm was read using a SpectraMax 340PC384 microplate reader. Using the absorbances determined, the following equation was used to calculate percent cytotoxicity:
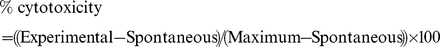



### Apoptosis Assay

Cells (∼10^5^ per well) were plated onto a flat bottom 96-well tissue culture plate and incubated in their normal media containing IgG2b (5 µg/ml), SEAM 3 (5 µg/ml), DMSO or 0.1 µM staurosporine at 37°C for 12 or 24 hours. Using a Vybrant Apoptosis Assay Kit #3 (Invitrogen) cells were stained with FITC Annexin V and propidium iodide according to the manufacturer's instructions. Binding was determined using the Guava EasyCyte flow cytometer and the data analyzed using FlowJo (TreeStar).
